# Conflict Experience Regulates the Neural Encoding of Cognitive Conflict

**DOI:** 10.3390/brainsci13060880

**Published:** 2023-05-30

**Authors:** Hui Jiang, Chaozheng Huang, Zekai Li, Qiuyun Wang, Weisong Liang, Aibao Zhou

**Affiliations:** 1 School of Psychology, Northwest Normal University, Lanzhou 730070, China; jhui119@163.com (H.J.);; 2 School of Judicial Police, Gansu University of Political Science and Law, Lanzhou 730070, China; hcz13320724256@163.com

**Keywords:** conflict experience, cognitive control, neural encoding, multivoxel pattern analysis

## Abstract

Cognitive control is adaptive in that it rapidly adjusts attention in response to changing contexts and shifting goals. Research provides evidence that cognitive control can rapidly adjust attention to focus on task-relevant information based on prior conflict experience. Neural encoding of goal-related information is critical for goal-directed behaviour; however, the empirical evidence on how conflict experience affects the encoding of cognitive conflict in the brain is rather weak. In the present fMRI study, a Stroop task with different proportions of incongruent trial was used to investigate the neural encoding of cognitive conflict in the environment with changing conflict experience. The results showed that the anterior cingulate cortex, dorsolateral prefrontal cortex, and intraparietal sulcus played a pivotal role in the neural encoding of cognitive conflict. The classification in anterior cingulate cortex was significantly above chance in the high-proportion, moderate-proportion, and low-proportion conflict conditions conducted separately, suggesting that neural encoding of cognitive conflict in this region was not altered based on proportion of conflict. The dorsolateral prefrontal cortex and intraparietal sulcus showed significant above-chance classification in the moderate-proportion and low-proportion conflict conditions, but not in the high-proportion conflict condition. These findings provide direct evidence that conflict experience modulates the neural encoding of cognitive conflict.

## 1. Introduction

The adage, ‘Learn from one’s mistakes’, is often applied to daily life and learning. People frequently adjust their current behaviour based on their experiences to improve the completion of goal-oriented tasks. Cognitive control serves as the foundation of goal-directed behaviour via effectively mobilising attentional resources to enhance target stimuli processing and suppress the interference of distracting stimuli based on prior experience [1,2,3,4,5]. For example, if a driver misses a highway exit because of a billboard distraction, they can use top-down attentional adjustments to suppress the interference caused by the billboard and focus on the highway exit sign when approaching the next exit. Therefore, cognitive control can flexibly adjust attention based on previous experiences through adapting to constantly changing environments and goals.

The Stroop task is a commonly used approach to study adaptive cognitive control, in which individuals are assessed for their ability to adjust cognitive control in a top-down manner according to conflict experience via manipulating the proportion of conflict trials in different blocks [4,6,7,8,9]. Compared to blocks with a low conflict ratio (20% incongruent trials), individuals in blocks with a high conflict ratio (80% incongruent trials) can acquire more conflict resolution experience. Based on this experience, individuals can mobilise attentional resources in a top-down manner to enhance target stimuli processing, promote conflict resolution, and, thus, exhibit smaller interference effects [2,3,8]. Functional magnetic resonance imaging (fMRI) studies found that compared to low-conflict ratio blocks, individuals processing cognitive conflict in high-conflict ratio blocks show significantly reduced activation in the dorsal lateral prefrontal cortex (DLPFC), anterior insula, anterior cingulate cortex (ACC), lateral parietal lobe, and inferior frontal gyrus, all of which are frontoparietal-related brain regions [9,10,11,12,13,14].

Previous studies showed that the processing of cognitive control typically involves the means through which the brain encodes and processes conflict [15,16]. Existing fMRI studies used univariate analysis to examine the relative changes in blood oxygenation signals within single voxels during congruent and incongruent trials, exploring the activation changes in conflict-related brain areas, such as the frontoparietal lobe, during cognitive conflict processing. However, increased activation of a specific brain region does not necessarily indicate that the relevant information can be encoded more effectively in that region [17]. Notably, neural encoding based on goal-related information is crucial for goal-directed behaviour; individuals encode goal-relevant information and subsequently mobilise attentional resources to enhance target stimulus processing [18,19]. Previous studies showed that compared to univariate activation analysis, multivariate pattern analysis (MVPA) is more sensitive in detecting subtle multivoxel pattern changes in fMRI data and can decode information related to experimental conditions from voxel activation patterns [20,21]. For example, Haxby et al. [22] used univariate analysis and found no significant differences in the activation of the ventral temporal lobe when individuals saw patterns of ‘shoes’ and ‘bottles’. However, MVPA could decode the pattern changes between voxels in the ventral temporal lobe and effectively predict whether the individual saw ‘shoes’ or ‘bottles’. Only a few studies used MVPA to examine the brain’s representation of cognitive conflict, consistently finding that the medial frontal cortex is an important brain region for characterizing cognitive conflict. For instance, Jiang and Egner used MVPA to examine the representation of stimulus-conflict (Stroop task) and ideomotor-conflict (Simon task) in the brain and found that the brain regions responsible for representing stimulus-conflict (Stroop task) and ideomotor-conflict (Simon task) differed. In particular, stimulus-conflict-specific representations were found bilaterally in dorsolateral, ventrolateral, and ventromedial prefrontal cortices, as well as in the parietal and occipito-temporal regions, while the ideomotor-conflict-specific representations were found mainly in the rostral anterior cingulate cortex (ACC), the left lingual gyrus, the left parahippocampal gyrus, and the bilateral calcarine sulci. The stimulus-conflict (Stroop task) and ideomotor-conflict (Simon task) were jointly characterised in the medial frontal cortex [23]. Kragel et al. combined multiple tasks containing cognitive conflict (e.g., working memory, response inhibition, and conflict processing tasks) and observed that these tasks were jointly represented in the medial frontal cortex, specifically the anterior supplementary motor area and anterior cingulate gyrus [24]. Vermeylen et al. used the Stroop task to find that colour–word conflict was mainly characterised in the medial frontal cortex [25]. These findings only suggest a stable representation of cognitive conflict in the brain. However, our processing of information is influenced by the external environment and internal states, yet no previous studies explored whether the brain’s representation of cognitive conflict is also modulated based on conflict experiences.

In summary, in this study, we aim to examine the neural mechanisms of conflict ratio regulation on cognitive control using the Stroop task through setting high-, medium-, and low-conflict ratio conditions. We employ univariate activation analysis to investigate how the conflict ratio modulates the brain’s processing of conflict and verify the reliability of existing research results. Furthermore, using MVPA and calculating the spatial patterns formed via multiple voxels, we analyse the differences between the brain’s encoding of cognitive conflict across different conflict ratio blocks.

## 2. Methods

### 2.1. Participants

A total of 32 college students participated in the experiment. All participants were right-handed, reported normal or corrected vision, normal colour vision, and had no history of neurological or psychological/cognitive impairments. Data from two participants were excluded because of excessive head movement (>2 mm) during the scanning process. Therefore, final valid data were collected from 30 participants (14 females; age, 18–26; mean ± SD, 21.54 ± 2.19 years).

All participants completed the ‘Scanning Participant Checklist’ before the experiment, and those who met the screening requirements were informed of the experimental procedures in detail and signed informed consent forms. The experiment protocol was approved by the Ethics Board of Northwest Normal University.

### 2.2. Tasks and Procedures

The classic Stroop colour–word paradigm was used in the experiments. The stimulus materials were the Chinese characters for ‘red’ (255, 0, 0), ‘green’ (0, 255, 0), ‘yellow’ (RGB: 255, 255, 0), and ‘blue’ (RGB: 0, 0, 255), with the characters written in red, green, yellow, and blue, respectively. Each trial began with a ‘+’ fixation point for 500 ms, followed by the presentation of the stimulus material for 500 ms and a blank screen for 2000 ms. Participants were asked to ignore the meaning of the character (task-irrelevant information) and quickly and accurately judge the colour of the character (task-relevant information). During the experiment, participants lay in the MRI scanner, responding with the middle finger of their left hand for red, the index finger of their left hand for green, the index finger of their right hand for yellow, and the middle finger of their right hand for blue.

**Figure 1:**
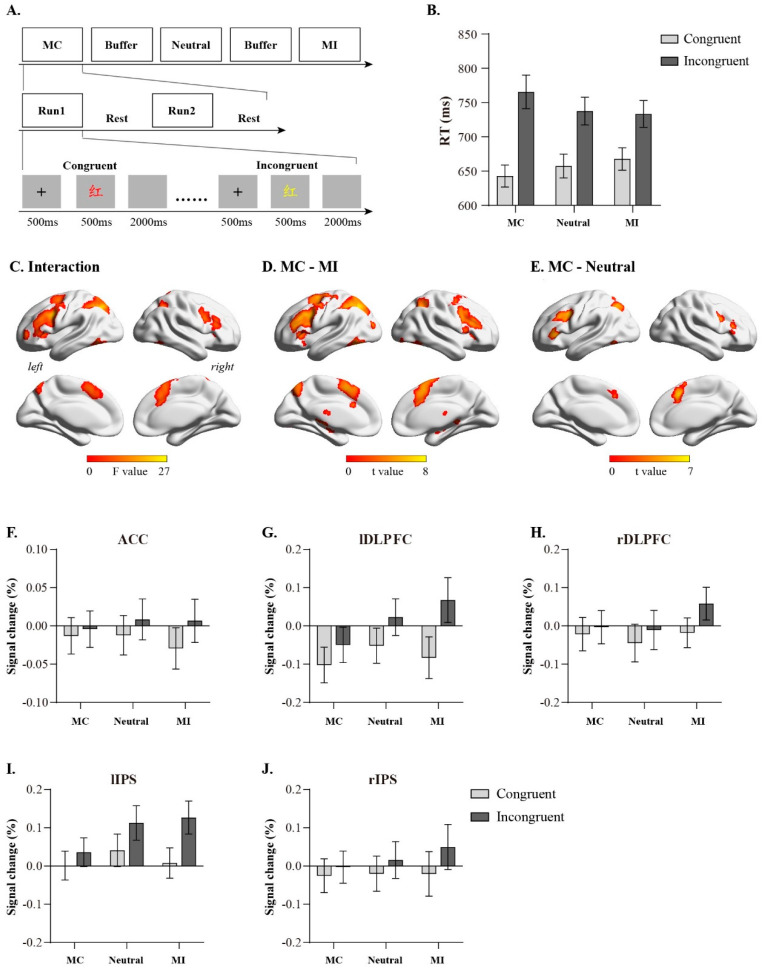
Experimental design and results of univariate analysis. (**A**): experimental procedure (The Chinese character “红” means red); (**B**): behavioral results; (**C**): brain regions involved in interaction between congruency and congruency proportion; (**D**): brain activation on MC condition compared to MI condition; (**E**): brain activation on MC condition compared to neutral condition; (**F**–**J**): Detailed activation profiles for representative brain regions. Error bars indicate standard error.

The experimental tasks included three types of blocks with varying conflict ratios: most congruent (MC), neutral, and most incongruent (MI). MC blocks had 25% incongruent and 75% congruent trials, neutral blocks had 50% incongruent and 50% congruent trials, and MI blocks had 75% incongruent and 25% congruent trials. Each conflict ratio condition included 2 blocks, with each block containing 120 trials, for a total of 240 trials per conflict ratio condition. Previous studies found that the proportional effect is long-lasting, and the formed effect can interfere with subsequent stimulus processing [26]. To eliminate the interference between different types of blocks, a buffer block was set between different conflict ratio conditions (Figure 1A). The buffer block contained 80 trials, including 40 incongruent trials and 40 congruent trials. A 2-min rest was provided after each block, and the presentation order of the three conflict ratio conditions was balanced across participants.

### 2.3. fMRI Data Acquisition

fMRI data were collected using a Siemens 3T scanner (Siemens Magnetom Trio TIM, Erlangen, Germany), with echo planar imaging (EPI) parameters set for functional images as follows: repetition time = 1500 ms; echo time = 30 ms; flip angle = 90°; the field of view = 192 mm; acquisition matrix = 64 × 64; interleaved scanning = 24 axial slices; and slice thickness = 3 mm. High-resolution T1-weighted brain structural images of the participants were also acquired using a fast gradient echo pulse sequence, which had specific parameters: repetition time = 1900 ms; echo time = 2.52 ms; flip angle = 9°; and matrix = 256 × 256.

### 2.4. fMRI Data Pre-Processing

Data pre-processing and analysis were both based on the SPM12 software (http://www.fil.ion.ucl.ac.uk/spm/spm8 accessed on 23 July 2022). Pre-processing first involved deleting the data from the first five time points of each run to ensure that the magnetic resonance signal reached a steady state. Next, slice timing correction was applied to adjust the scanning sequence of the acquired functional images across different slices. Head motion correction (realignment) was performed to estimate and correct for head motion in the functional image data to avoid significant impacts on data quality. The participants’ structural T1-weighted images were then registered to the averaged functional image data and further segmented into grey matter, white matter, and cerebrospinal fluid. Finally, spatial normalisation was performed to normalise the registered data to the Montreal Neurological Institute coordinate system, with a voxel size of 3 × 3 × 3 mm. The last step of pre-processing involved smoothing with a Gaussian spatial filter to process the already normalised EPI images and reduce errors and individual differences in brain structure introduced via the normalisation process. The size of the smoothing kernel, which was measured in full width at half maximum (FWHM), was 6 × 6 × 6 mm.

### 2.5. Univariate Activation Analysis

A general linear model (GLM) was used to detect brain activity induced under different experimental conditions. The model included six blocks, with each block containing three regressors: congruent, incongruent, and erroneous trials. These regressors were then convoluted with the standard hemodynamic response function (HRF). Additionally, to control for the effects of head motion, six head motion parameters (three translational and three rotational parameters) were included in the model as unrelated regressors. The model adopted a high-pass filter of 1/128 Hz, and the restricted maximum likelihood method was used for parameter estimation.

Individual and group level statistical analyses were conducted using GLM according to the purpose of the experiment. Firstly, single-sample *t*-tests were performed for the six conditions [2 (trial congruency: congruent trials, incongruent trials) × 3 (conflict ratio conditions: MC, Neutral, MI)] to obtain the activation statistics map for each condition. Subsequently, in the group analyses, a full factorial design was used to conduct a repeated measures analysis of variance (ANOVA) on the 2 (trial congruency: congruent trials, incongruent trials) × 3 (conflict ratio: MC, Neutral, MI) repeated measures ANOVA factors, which examined the brain activity induced through the interactions of experimental conditions. Finally, [(MC incongruent trials–MC congruent trials) − (MI incongruent trials–MI congruent trials)] was used to investigate the neural activity differences between MC and MI conditions during conflict processing, while [(MC incongruent trials–MC congruent trials) − (Neutral incongruent trials–Neutral congruent trials)] was used to investigate the neural activity differences between MC and neutral conditions during conflict processing. All analysis results in this study were corrected for multiple comparisons (false discovery rate (FDR), *p* < 0.05, k > 30). Additionally, to further understand how conflict experience regulates neural activity during conflict processing in each brain region, the signal change values of the brain regions corrected for multiple comparisons in the above analyses were extracted to perform the repeated measures ANOVA on the 2 (trial congruency: congruent trials, incongruent trials) × 3 (conflict ratio: MC, Neutral, MI) factors.

### 2.6. Multivoxel Pattern Analysis

In this study, we used fMRI data that were not standardised or smoothed in space and performed multi-voxel pattern analysis in the individual’s native space. Before multivoxel pattern analysis occurred, activation maps for each valid trial were calculated based on GLM [27]. Specifically, each trial was defined as an independent regressor using the stick function, and these regressors were convolved with the standard HRF. To control for head motion effects, six head motion parameters (three translational and three rotational) were included as unrelated regressors in the model. The model used a high-pass filter of 1/128 Hz. Finally, beta maps were obtained for each trial, which reflected the intensity of neural response induced using different trial signals, and further used for multivoxel pattern analysis.

Using the Decoding Toolbox [28] and a linear support vector machine (with a parameter setting of c = 1), we employed a searchlight multivoxel pattern analysis method to identify brain regions capable of decoding cognitive conflicts. A whole-brain grey matter mask was used in the searchlight multivoxel pattern analysis to limit the searchlight search area. At each step, a voxel was selected as the centre, and the data from other voxels within a 4-voxel radius of the centre were combined to construct the feature vector for classification. The resulting classification accuracy was assigned to the central voxel, and this procedure was repeated for every voxel in the whole brain. We trained classifiers under MC, neutral, and MI conditions, classifying congruent and incongruent trials. When training each classifier, we adopted a leave-one-run-out cross-validation method, with one of the two blocks used as the training set and the other as the test set. Given the differences in the experimental setting and individual differences in participant responses, the number of congruent and incongruent trials often differed across different conflict ratios, which could lead to classifier bias. To avoid such bias, an equal number of trials from each category were randomly selected for training and testing in each iteration (e.g., if the number of congruent trials exceeded that of incongruent trials, an equal number of congruent trials as the number of incongruent trials would be randomly selected, and vice versa). This process was repeated 10 times for each conflict ratio, and the average classification accuracy for each voxel was calculated to obtain the whole-brain classification accuracy map. Next, using the warping parameters obtained during pre-processing, we normalised the individual classification accuracy map and smoothed the data with a 6 mm FWHM Gaussian smoothing kernel. We then conducted a second-level group analysis using one-sample *t*-tests on the smoothed and normalised accuracy map, identifying voxels with significantly higher classification accuracies than the chance level (0.5) at the group level (results were corrected for multiple comparisons: FDR, *p* < 0.05, k > 30). To further investigate how conflict experience regulates the representation of conflict in each brain region, we extracted the classification accuracy of cognitive control-related brain regions under each conflict ratio condition for statistical analysis.

## 3. Results

### 3.1. Behavioural Results

We excluded the first trial (0.83%), erroneous trials, post-error trials, and trials with reaction times beyond ± 3 standard deviations of the mean under each condition (10.4%) from each block. A 2 (trial congruency: congruent trials, incongruent trials) × 3 (conflict ratio: MC, Neutral, MI) repeated measures ANOVA was conducted on reaction times and accuracy rates. The analysis of reaction times (Figure 1B, Table 1) revealed a significant main effect of trial congruency (*F*(1, 29) = 152.59, *p* < 0.001, η^2^ = 0.84), with congruent trials (mean ± SEM: 655 ± 15 ms) significantly faster than incongruent trials (745 ± 20 ms). The main effect of the conflict ratio was not significant (*F*(2, 58) = 0.21, *p* = 0.81, η^2^ = 0.007). A significant interaction existed between the trial congruency and the conflict ratio (*F*(2, 58) = 26.81, *p* < 0.001, η^2^ = 0.48). Simple effects analysis demonstrated that under congruent trial conditions, the reaction time of the MC condition was significantly shorter than that of the MI condition (*p* = 0.01); under incongruent trial conditions, the reaction time of the MC condition was significantly longer than that of the MI condition (*p* = 0.02). Further analysis revealed that the interference effect under the MC condition (incongruent trial reaction time–congruent trial reaction time: 123 ms) was greater than the interference effect under the neutral condition (*t*(29) = 4.33, *p* < 0.001, Cohen’s d = 0.79), the interference effect under the neutral condition (incongruent trial reaction time–congruent trial reaction time: 80 ms) was greater than the interference effect under the MI condition (65 ms, *t*(29) = 2.31, *p* = 0.03, Cohen’s d = 0.42), and the interference effect under the MC condition was greater than the interference effect under the MI condition (*t*(29) = 7.34, *p* < 0.001, Cohen’s d = 1.34); these results indicate that the conflict ratio can modulate trial congruency effects.

Analysis based on accuracy showed no significance in the main effects of trial congruency, conflict ratio, or their interaction (main effect of conflict ratio: *F*(2, 58) = 0.88, *p* = 0.42, η^2^ = 0.29; main effect of trial congruency: *F*(1, 29) = 3.56, *p* = 0.07, η^2^= 0.11; interaction effect: *F*(2, 58) = 1.73, *p* = 0.19, η^2^ = 0.06)).

### 3.2. fMRI Results

Using univariate activation analysis, the fMRI data were first subjected to a 2 (trial congruency: congruent trials, incongruent trials) × 3 (conflict ratio: MC, Neutral, MI) repeated measures ANOVA. The results revealed that the interaction between the trial congruency and the conflict ratio was mainly manifested in the activation of the bilateral dorsolateral prefrontal cortex, bilateral intraparietal sulcus (IPS), and ACC (Figure 1C); further analysis via [(MC incongruent trial–MC congruent trial)–(MI incongruent trial–MI congruent trial)] found that MC significantly enhanced the activation of the bilateral dorsolateral prefrontal cortex, bilateral IPS, and ACC during conflict processing compared to MI (Figure 1D). [(MC incongruent trial–MC congruent trial)–(Neutral incongruent trial–Neutral congruent trial)] revealed that MC significantly enhanced the activation of the bilateral dorsolateral prefrontal cortex, left IPS, and ACC during conflict processing compared to neutral. We then extracted the signal change values under each condition, such as the ACC (10, 24, 26), right dorsolateral prefrontal cortex (DLPFC; 44, 10, 28), right IPS (34, −56, 52), left DLPFC (−46, 12, 32), and left IPS (−26, −62, 42), from brain regions. The results showed that the main effects of the trial congruency were significant in these brain regions (ACC: *F*(1, 29) = 53.61, *p* < 0.001, η^2^ = 0.65; right DLPFC: *F*(1, 29) = 46.35, *p* < 0.001, η^2^ = 0.62; right IPS: *F*(1, 29) = 54.18, *p* < 0.001, η^2^ = 0.65; left DLPFC: *F*(1, 29) = 100.75, *p* < 0.001, η^2^ = 0.78; left IPS: *F*(1, 29) = 113.58, *p* < 0.001, η^2^ = 0.80), whereas the main effects of conflict ratio were not significant (*ps* > 0.05). The interaction between the congruency and the conflict ratio was significant (CC: *F*(2, 58) = 6.15, *p* = 0.004, η^2^ = 0.18; right DLPFC: *F*(2, 58) = 15.91, *p* < 0.001, η^2^ = 0.35; right IPS: *F*(2, 58) = 8.19, *p* < 0.001, η^2^ = 0.22; left DLPFC: *F*(2, 58) = 28.99, *p* < 0.001, η^2^ = 0.50; left IPS: *F*(2, 58) = 32.72, *p* < 0.001, η^2^ = 0.53). Simple effect analysis revealed no significant differences among MC, Neutral, and MI conditions for either congruent or incongruent trials (*ps* > 0.05). Further analysis revealed that the conflict-processing-related activation levels (signal change in incongruent trial–signal change in congruent trial) in these brain regions under the MC condition were greater than those under the MI condition (ACC: *t*(29) = 3.08, *p* = 0.004; right DLPFC: *t*(29) = 4.79, *p* < 0.001; right IPS: *t*(29) = 3.68, *p* < 0.001; left DLPFC: *t*(29) = 6.88, *p* < 0.001; left IPS: *t*(29) = 7.09, *p* < 0.001), and the activation levels under the neutral condition were greater than those under the MI condition (ACC: *t*(29) = 2.67, *p* = 0.01; right DLPFC: *t*(29) = 4.21, *p* < 0.001; right IPS: *t*(29) = 2.89, *p* = 0.007; left DLPFC: *t*(29) = 5.36, *p* < 0.001; left IPS: *t*(29) = 4.53, *p* < 0.001). These results indicate that the conflict ratio can modulate the activation of brain regions, such as the ACC, right DLPFC, right IPS, left DLPFC, and left IPS, during conflict processing.

**Figure 2:**
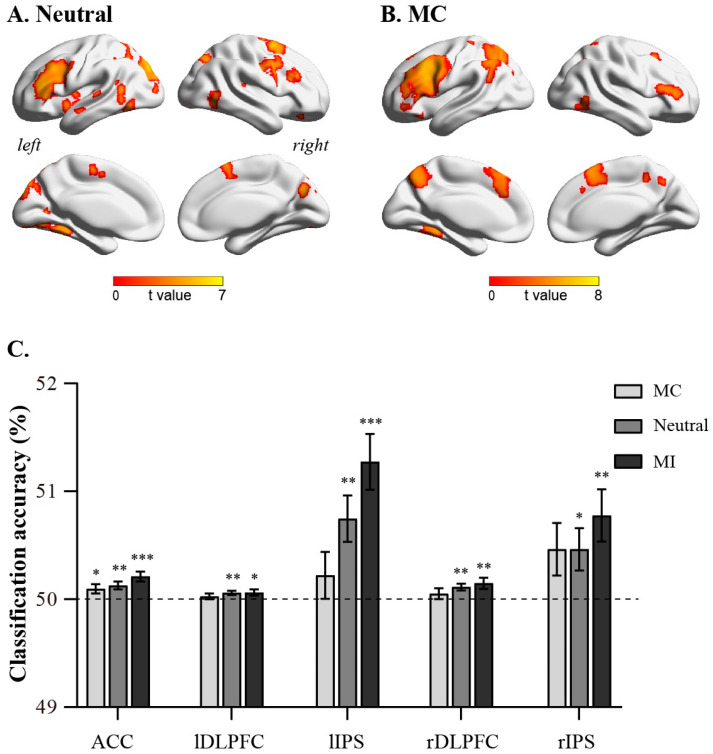
Results of multivariate pattern analysis. (**A**): nrain regions encoding cognitive conflict in neutral condition; (**B**): brain regions encoding cognitive conflict in MC condition. (**C**): classification accuracy of ACC, left DLPFC, left IPS, right DLPFC, and right IPS in MC, neutral, and MI conditions. Error bars indicate standard error. Significance markings for individual bars indicate whether coding of that task’s features was significantly greater than chance (one-sample *t*-test against chance, 50%) in that region in that condition. (* *p* < 0.05, ** *p* < 0.01, *** *p* < 0.001).

The searchlight-based multivoxel pattern analysis method showed that under MC and neutral conditions, brain regions, such as the ACC, bilateral DLPFC, and bilateral IPS, can significantly decode congruent and incongruent trials (Figure 2A,B). However, no brain regions that could significantly decode congruent and incongruent trials were found under the MI condition. We then extracted the classification accuracies of ACC (10, 24, 26), right DLPFC (44, 10, 28), right IPS (34, −56, 52), left DLPFC (−46, 12, 32), and left IPS (−26, −62, 42) (Figure 2C). The results demonstrated that the classification accuracies of these brain regions were significantly greater than random levels under MC (ACC: *t*(29) = 4.64, *p* < 0.001; left DLPFC: *t*(29) = 2.12, *p* = 0.04; right IPS: *t*(29) = 3.20, *p* = 0.003; right DLPFC: *t*(29) = 2.88, *p* = 0.007; left IPS: *t*(29) = 4.94, *p* < 0.001) and neutral conditions (ACC: *t*(29) = 3.48, *p* = 0.002; left DLPFC: *t*(29) = 2.83, *p* = 0.008; right IPS: *t*(29) = 2.36, *p* = 0.025; right DLPFC: *t*(29) = 3.62, *p* < 0.001; left IPS: *t*(29) = 3.48, *p* = 0.002). However, under the MI condition, only the classification accuracy of the ACC (*t*(29) = 2.26, *p* = 0.032) was significantly greater than random levels, while the classification accuracies of the right DLPFC, right IPS, left DLPFC, and left IPS did not significantly differ from random levels (*ps* > 0.05). Moreover, the classification accuracies of these brain regions did not significantly differ among the MC, Neutral, and MI conditions (*ps* > 0.05).

## 4. Discussion

In this study, we investigated the neural mechanisms of conflict experience in regulating cognitive control through manipulating the ratio of conflict trials in different blocks of the Stroop task with the fMRI technology. Behavioural results showed that as the ratio of incongruent trials increased in the blocks, the interference effect of the Stroop task gradually decreased, indicating that individuals can adjust their cognitive control based on conflict experience, enhancing their conflict resolution abilities. Univariate activation analysis results demonstrated that conflict ratio primarily modulated the neural activity of the ACC, bilateral DLPFC, and bilateral IPS during conflict processing. Specifically, compared to high-conflict ratio blocks, low-conflict ratio blocks significantly enhanced the activation levels of the ACC, bilateral DLPFC, and bilateral IPS during conflict resolution. Moreover, multivoxel pattern analysis results revealed that the ACC, bilateral DLPFC, and bilateral IPS were key brain regions encoding cognitive conflict. Among them, the ACC could significantly decode congruent and incongruent trials under all three conflict ratio conditions; however, the bilateral DLPFC and bilateral IPS could not decode congruent and incongruent trials under high-conflict radio conditions, but could significantly decode these trials under low- and medium-conflict ratio conditions.

In terms of behaviour, consistent with previous research on conflict ratio effects [9,13], the higher the conflict ratio in blocks, the smaller the interference effect of the Stroop task. This finding suggests that the representation and maintenance of experience information in the working memory system are crucial for goal-directed behaviour. Individuals can effectively predict cognitive needs based on experience and current environmental information, and enhance top-down attention to task-related stimuli [2,3,4]. Notably, most trials experienced by individuals in high-conflict ratio blocks were incongruent trials, where task-irrelevant information (meaning of the character) and task-relevant information (colour of the character) often contradict each other. To effectively complete the judgement response to the target stimulus and inhibit the interference of task-irrelevant information on task-relevant information, participants enhanced top-down attention to the colour of the characters and weakened attention to their meaning during the experiment. Therefore, the interference effect in high-conflict ratio blocks is smaller than in medium- and low-conflict ratio blocks. In contrast, in low-conflict ratio blocks, most trials experienced by participants were congruent trials. As character colour and meaning are identical under congruent trials and processing of character meaning is more automatic than character colour, to induce a faster keypress response, individuals depend on character meaning for a faster judgement of character colour. However, when facing conflicts, participants needed to exert more effort to refocus attention on character colour, while ignoring the interference of character meaning. Therefore, compared to medium- and high-conflict ratio conditions, low-conflict ratio blocks exhibit a greater interference effect.

Given the need for more cognitive effort to focus attention on the target stimulus and suppress the interference of distractor stimuli in low-conflict ratio conditions, the activation of cognitive control-related brain regions, such as the ACC, DLPFC, and IPS, is significantly enhanced in low-conflict ratio conditions compared to high-conflict ratio conditions. Among these regions, the ACC is mainly responsible for conflict monitoring, while the DLPFC and IPS are mainly responsible for conflict resolution [29,30,31]. Moreover, the cognitive control network, which comprises the ACC, DLPFC, and IPS, is commonly activated via events with an increase in information uncertainty [32]. In the present study, fewer conflicting trials in the low-conflict ratio block made it more difficult for subjects to predict whether the next trial would be a conflicting trial; therefore, the uncertainty was higher. Accordingly, significantly higher cognitive control network activation was observed in the low-conflict ratio block than in the high-conflict ratio block.

In addition, researchers believe that in conflict ratio experiments, individuals tend to adopt proactive control strategies in high-conflict blocks, i.e., predict the upcoming responses based on the cue information and selectively process task-relevant cue information in a top-down manner [8,13,33]. In contrast, in low-conflict blocks, individuals are more likely to adopt reactive control strategies, i.e., they flexibly use the instantly emerging task-relevant information to resolve conflicts [8,13,34]. Compared to proactive control strategies, reactive control strategies are primarily related to the activation of the frontoparietal network (FPN) [34,35,36,37,38], with the DLPFC and IPS being the core brain regions that constitute the FPN and are responsible for the initiation and adjustment of cognitive control [39]. Based on the above analysis, the activation results of this study validate the existing research findings, indicating that conflict ratio effectively resolves conflicts via regulating the activation of cognitive control-related brain regions, such as the ACC, DLPFC, and IPS, further demonstrating the reliability of the study data and results.

The brain’s representation of cognitive conflicts is one of the crucial processes in cognitive control processing [15]. In this study, we employed multivoxel pattern analysis and found that the ACC, DLPFC, and IPS are key brain regions that encode cognitive conflicts, and these regions exhibit different neural response patterns in terms of conflict encoding and activation results. Based on univariate activation analysis, the ACC, DLPFC, and IPS displayed different intensities of activation between different conflict ratio conditions. However, based on multivoxel pattern analysis, the encoding differences of cognitive conflicts in these brain regions between different conflict ratio conditions were not significant, indicating that conflict representation is different from conflict processing detected via univariate activation analysis. During the process of conflict representation, the ACC is mainly responsible for monitoring environmental changes and the emergence of conflicts [25,40], as well as predicting demands for control [3,41]. Therefore, we found that the ACC can significantly decode conflicts under all three conflict ratio conditions, although no significant difference exists in decoding accuracy under the three conflict ratio conditions. The DLPFC and IPS are involved in the encoding of task-relevant information and demand control [3,18,25,42]. Compared to low- and medium-conflict ratio blocks, individuals in high-conflict ratio conditions can enhance their attention to the colour of Chinese characters in a top-down manner, perceive minimal cognitive conflicts, and have a smaller demand for control of distractor stimuli, which may result in the DLPFC and IPS being unable to decode congruent and incongruent trials. In contrast, in low- and medium-conflict ratio blocks, incongruent trials require more cognitive effort to suppress the interference of distractor stimuli with the target stimuli compared to congruent trials, allowing the DLPFC and IPS to significantly decode congruent and incongruent trials.

In summary, we investigated how conflict experiences regulate cognitive control via manipulating the ratio of conflict trials in different blocks of the Stroop task. On one hand, the results confirmed findings from existing conflict ratio-related research, which showed that individuals’ response times in resolving conflicts were significantly greater in low-conflict ratio blocks compared to high-conflict ratio blocks, and found that low-conflict ratio blocks exhibited stronger activation responses in the ACC, DLPFC, and IPS compared to high-conflict ratio blocks. On the other hand, we employed multivoxel pattern analysis to examine how conflict ratio affects conflict representation and found that the ACC, DLPFC, and IPS are key brain regions involved in representing cognitive conflicts. Among these regions, the ACC’s representation of conflicts does not change with variations in conflict ratios, whereas the DLPFC and IPS can significantly represent conflicts under medium- and low-conflict ratio conditions but not under high-conflict ratio conditions. These results suggest that conflict processing and conflict representation are two independent processing mechanisms, and conflict experiences promote conflict resolution through influencing both processes.

## Figures and Tables

**Table 1:** Response time (ms) and accuracy (%) for task conditions.

	MC		Neutral		MI	
	Congruency	Incongruent	Congruency	Incongruent	Congruency	Incongruent
Accuracy (%)	84 ± 12	87 ± 7	86 ± 10	87 ± 6	86 ± 10	87 ± 7
Response time (ms)	642 ± 87	765 ± 134	657 ± 95	738 ± 110	668 ± 89	733 ± 106

## Data Availability

The datasets of the current study are available from the corresponding authors on reasonable request.

## References

[1] Chiu Y.C., Egner T. (2019). Cortical and subcortical contributions to context-control learning. Neurosci. Biobehav. Rev..

[2] Jiang J., Heller K., Egner T. (2014). Bayesian modeling of flexible cognitive control. Neurosci. Biobehav. Rev..

[3] Jiang J., Beck J., Heller K., Egner T. (2015). An insula-frontostriatal network mediates flexible cognitive control by adaptively predicting changing control demands. Nat. Commun..

[4] Suh J., Bugg J.M. (2021). The shaping of cognitive control based on the adaptive weighting of expectations and experience. J. Exp. Psychol. Learn. Mem Cogn..

[5] Cai W., Chen T., Ryali S., Kochalka J., Li C.S., Menon V. (2016). Causal Interactions Within a Frontal-Cingulate-Parietal Network During Cognitive Control: Convergent Evidence from a Multisite-Multitask Investigation. Cereb. Cortex..

[6] Braem S., Bugg J.M., Schmidt J.R., Crump M.J.C., Weissman D.H., Notebaert W., Egner T. (2019). Measuring Adaptive Control in Conflict Tasks. Trends Cogn. Sci..

[7] Yang G., Xu H., Li Z., Nan W., Wu H., Li Q., Liu X. (2021). The congruency sequence effect is modulated by the similarity of conflicts. J. Exp. Psychol. Learn. Mem. Cogn..

[8] Spinelli G., Lupker S.J. (2021). Proactive control in the Stroop task: A conflict-frequency manipulation free of item-specific, contingency-learning, and color-word correlation confounds. J. Exp. Psychol. Learn. Mem. Cogn..

[9] Blais C., Bunge S. (2010). Behavioral and neural evidence for item-specific performance monitoring. J. Cogn. Neurosci..

[10] Menon V.D., Esposito M. (2021). The role of PFC networks in cognitive control and executive function. Neuropsychopharmacology.

[11] Friedman N.P., Robbins T.W. (2021). The role of prefrontal cortex in cognitive control and executive function. Neuropsychopharmacology.

[12] Xia T., Li H., Wang L. (2016). Implicitly strengthened task-irrelevant stimulus-response associations modulate cognitive control: Evidence from an fMRI study. Hum. Brain Mapp..

[13] Aben B., Calderon C.B., Van der Cruyssen L., Picksak D., Van den Bussche E., Verguts T. (2019). Context-dependent modulation of cognitive control involves different temporal profiles of fronto-parietal activity. Neuroimage.

[14] Feng C., Becker B., Huang W., Wu X., Eickhoff S.B., Chen T. (2018). Neural substrates of the emotion-word and emotional counting Stroop tasks in healthy and clinical populations: A meta-analysis of functional brain imaging studies. Neuroimage.

[15] Freund M.C., Etzel J.A., Braver T.S. (2021). Neural Coding of Cognitive Control: The Representational Similarity Analysis Approach. Trends Cogn. Sci..

[16] Wood J.N., Grafman J. (2003). Human prefrontal cortex: Processing and representational perspectives. Nat. Rev. Neurosci..

[17] Riggall A.C., Postle B.R. (2012). The relationship between working memory storage and elevated activity as measured with functional magnetic resonance imaging. J. Neurosci..

[18] Etzel J.A., Cole M.W., Zacks J.M., Kay K.N., Braver T.S. (2016). Reward Motivation Enhances Task Coding in Frontoparietal Cortex. Cereb. Cortex..

[19] Manelis A., Reder L.M. (2015). He who is well prepared has half won the battle: An FMRI study of task preparation. Cereb. Cortex..

[20] Norman K.A., Polyn S.M., Detre G.J., Haxby J.V. (2006). Beyond mind-reading: Multi-voxel pattern analysis of fMRI data. Trends Cogn. Sci..

[21] Popal H., Wang Y., Olson I.R. (2019). A Guide to Representational Similarity Analysis for Social Neuroscience. Soc. Cogn. Affect. Neurosci..

[22] Haxby J.V., Gobbini M.I., Furey M.L., Ishai A., Schouten J.L., Pietrini P. (2001). Distributed and overlapping representations of faces and objects in ventral temporal cortex. Science.

[23] Jiang J., Egner T. (2014). Using neural pattern classifiers to quantify the modularity of conflict-control mechanisms in the human brain. Cereb. Cortex.

[24] Kragel P.A., Kano M., Van Oudenhove L., Ly H.G., Dupont P., Rubio A., Delon-Martin C., Bonaz B.L., Manuck S.B., Gianaros P.J. (2018). Generalizable representations of pain, cognitive control, and negative emotion in medial frontal cortex. Nat. Neurosci..

[25] Vermeylen L., Wisniewski D., González-García C., Hoofs V., Notebaert W., Braem S. (2020). Shared Neural Representations of Cognitive Conflict and Negative Affect in the Medial Frontal Cortex. J. Neurosci..

[26] Torres-Quesada M., Funes M.J., Lupianez J. (2013). Dissociating proportion congruent and conflict adaptation effects in a Simon-Stroop procedure. Acta Psychol..

[27] Mumford J.A., Turner B.O., Ashby F.G., Poldrack R.A. (2012). Deconvolving BOLD activation in event-related designs for multivoxel pattern classification analyses. NeuroImage.

[28] Hebart M.N., Gorgen K., Haynes J.D. (2014). The Decoding Toolbox (TDT): A versatile software package for multivariate analyses of functional imaging data. Front. Neuroinform..

[29] Botvinick M., Nystrom L.E., Fissell K., Carter C.S., Cohen J.D. (1999). Conflict monitoring versus selection-for-action in anterior cingulate cortex. Nature.

[30] Kerns J.G. (2006). Anterior cingulate and prefrontal cortex activity in an FMRI study of trial-to-trial adjustments on the Simon task. Neuroimage.

[31] Kerns J.G., Cohen J.D., MacDonald A.W., Cho R.Y., Stenger V.A., Carter C.S. (2004). Anterior cingulate conflict monitoring and adjustments in control. Science.

[32] Wu T., Schulz K.P., Fan J. (2021). Activation of the cognitive control network associated with information uncertainty. Neuroimage.

[33] Braver T.S. (2012). The variable nature of cognitive control: A dual mechanisms framework. Trends Cogn. Sci..

[34] Dosenbach N.U., Fair D.A., Miezin F.M., Cohen A.L., Wenger K.K., Dosenbach R.A., Fox M.D., Snyder A.Z., Vincent J.L., Raichle M.E. (2007). Distinct brain networks for adaptive and stable task control in humans. Proc. Natl. Acad. Sci. USA.

[35] Gratton C., Neta M., Sun H., Ploran E.J., Schlaggar B.L., Wheeler M.E., Petersen S.E., Nelson S.M. (2017). Distinct Stages of Moment-to-Moment Processing in the Cinguloopercular and Frontoparietal Networks. Cereb. Cortex..

[36] Dosenbach N.U., Fair D.A., Cohen A.L., Schlaggar B.L., Petersen S.E. (2008). A dual-networks architecture of top-down control. Trends Cogn. Sci..

[37] Braver T.S., Reynolds J.R., Donaldson D.I. (2003). Neural mechanisms of transient and sustained cognitive control during task switching. Neuron.

[38] Li Y., Wang Y., Yu F., Chen A. (2021). Large-scale reconfiguration of connectivity patterns among attentional networks during context-dependent adjustment of cognitive control. Hum. Brain Mapp..

[39] Corbetta M., Shulman G.L. (2002). Control of goal-directed and stimulus-driven attention in the brain. Nat. Rev. Neurosci..

[40] Behrens T.E., Woolrich M.W., Walton M.E., Rushworth M.F. (2007). Learning the value of information in an uncertain world. Nat. Neurosci..

[41] Jiang J., Brashier N.M., Egner T. (2015). Memory Meets Control in Hippocampal and Striatal Binding of Stimuli, Responses, and Attentional Control States. J. Neurosci..

[42] Jackson J.B., Feredoes E., Rich A.N., Woolgar A. (2021). Concurrent neuroimaging and neurostimulation reveals a causal role for dlPFC in coding of task-relevant information. Commun. Biol..

